# Multi-Head Self-Attention Model for Classification of Temporal Lobe Epilepsy Subtypes

**DOI:** 10.3389/fphys.2020.604764

**Published:** 2020-11-27

**Authors:** Peipei Gu, Ting Wu, Mingyang Zou, Yijie Pan, Jiayang Guo, Jianbing Xiahou, Xueping Peng, Hailong Li, Junxia Ma, Ling Zhang

**Affiliations:** ^1^Software Engineering College, Zhengzhou University of Light Industry, Zhengzhou, China; ^2^Department of Magnetoencephalography, Nanjing Brain Hospital Affiliated, Nanjing Medical University, Nanjing, China; ^3^School of Biomedical Engineering, Hubei University of Science and Technology, Xianning, China; ^4^Department of Computer Science and Technology, Tsinghua University, Beijing, China; ^5^Ningbo Institute of Information Technology Application, Chinese Academy of Sciences, Ningbo, China; ^6^Department of Electrical Engineering and Computer Science, University of Cincinnati, Cincinnati, OH, United States; ^7^School of Informatics, Xiamen University, Xiamen, China; ^8^Australian Artificial Intelligence Institute, Faculty of Engineering and Information Technology, University of Technology Sydney, Ultimo, NSW, Australia; ^9^The Perinatal Institute, Cincinnati Children’s Hospital Medical Center, Cincinnati, OH, United States

**Keywords:** TLE diagnosis, self-attention model, epilepsy classification, temporal lobe epilepsy detection, multi-head self-attention

## Abstract

As a long-standing chronic disease, Temporal Lobe Epilepsy (TLE), resulting from abnormal discharges of neurons and characterized by recurrent episodic central nervous system dysfunctions, has affected more than 70% of drug-resistant epilepsy patients across the world. As the etiology and clinical symptoms are complicated, differential diagnosis of TLE mainly relies on experienced clinicians, and specific diagnostic biomarkers remain unclear. Though great effort has been made regarding the genetics, pathology, and neuroimaging of TLE, an accurate and effective diagnosis of TLE, especially the TLE subtypes, remains an open problem. It is of a great importance to explore the brain network of TLE, since it can provide the basis for diagnoses and treatments of TLE. To this end, in this paper, we proposed a multi-head self-attention model (MSAM). By integrating the self-attention mechanism and multilayer perceptron method, the MSAM offers a promising tool to enhance the classification of TLE subtypes. In comparison with other approaches, including convolutional neural network (CNN), support vector machine (SVM), and random forest (RF), experimental results on our collected MEG dataset show that the MSAM achieves a supreme performance of 83.6% on accuracy, 90.9% on recall, 90.7% on precision, and 83.4% on F1-score, which outperforms its counterparts. Furthermore, effectiveness of varying head numbers of multi-head self-attention is assessed, which helps select the optimal number of multi-head. The self-attention aspect learns the weights of different signal locations which can effectively improve classification accuracy. In addition, the robustness of MSAM is extensively assessed with various ablation tests, which demonstrates the effectiveness and generalizability of the proposed approach.

## Introduction

Epilepsy, a chronic central nervous system disease, is typically caused by the repeated abnormal discharge of neurons and is characterized by symptoms that are sudden, periodic, and short-term. According to a recent survey (Beghi and Giussani, 2018), around 70 million people across the world are affected, of which, 90% are grouped in undeveloped areas. Epilepsy has a global occurrence rate of 6.38∼7.60% (Jette et al., 2017) and around 67.77 new cases for every hundred thousand people are found each year. The ones who fail to take control of epilepsy after drug treatments are known as drug-resistant epilepsy (DRE) (Wiebe, 2013), among which, temporal lobe epilepsy (TLE), a common type of epilepsy widely existing in young and elderly patients, accounts for around 70% (Mariani et al., 2019). With the growth of the population and advent of an aging society, it is inevitable that TLE will be of a great burden for human beings. Therefore, it is urgent to identify the subtype, cause, and inducement in the treatment of TLE. Though progress has been made through subjective analysis, traditional methods for imaging and clinical symptom assessment heavily rely on human experts, leading to a long diagnostic time. Moreover, subjective diagnostic results are often made from different experts, even for the same patient (Siuly and Li, 2015). Thus, it is hard to make medical decision using solely experts. Therefore, it is crucial to develop an efficient and objective TLE diagnosis method to support the treatment of TLE.

Typically, TLE can be categorized into three subtypes: simple partial seizure (SPS), complex partial seizure (), and these two types coexisting. The key difference between SPS and CPS lies in the disturbance of consciousness. In comparison with SPS, CPS is more likely to evolve into drug-resistant epilepsy, which denotes the ineffectiveness of drug treatments. On the contrary, taking antiepileptic drugs may result in side effects that affect the cognition and puberty development of the human brain, adding great emotional and economic burden to the patients and their families. An accurate diagnosis in the early stage of disease outbreak is fundamental to non-drug treatments, which avoids the dosage of drugs and further ensures a good quality of life for the patients. To this end, the medical community has put great effort into exploring the difference between the CPS and SPS brain networks and studying the treatments for different subtypes of brain network nodes.

Learning to classify these two subtypes accurately and objectively will benefit the clinical risk stratification and relieve the heavy dependency on human experts. In addition, by predicting the subtypes and taking active measures in advance, it also keeps the high-risk population with a conscious disorder from the risk of sudden death that results from the disturbance of consciousness after epileptic seizures.

In this study, we used collected magnetoencephalography (MEG) signals to classify the subtypes of temporal lobe epilepsy, as MEG has emerged as an non-invasive, reliable, fast, and easy-to-use technique to record functional activities of the brain (Englot et al., 2016; van Klink and Zijlmans, 2019; Shi et al., 2020). It has been observed that the spike-wave of epilepsy is indeed a time-dependent characteristic wave. MEG shows its great superiorities in acquiring the high-temporal resolution of data and spatial lateralization and localization (Liu et al., 2020). Therefore, compared with other tools, such as electroencephalogram (EEG) (Anastasiadou et al., 2019; Yao et al., 2019; Serna et al., 2020), MEG has been considered as an effective tool to diagnose epilepsy and find the location of cortical pathological activity or damage of epileptic foci (Burns et al., 2014).

Recent years have witnessed the emerging performance of deep learning in various research topics, including the study of epilepsy (Peng et al., 2019, 2020a,b; Niu et al., 2020). For example, Wu et al., 2018 proposed to deal with TLE lateralization based on MEG by transferring it into a series of binary classification problems. To that effect, the resting-state brain network is first employed to extract features of each participant, upon which the support vector machine (SVM) is built to achieve the classification of extracted features. Achilles et al., 2018 developed a non-invasive automatic system for monitoring epilepsies via resorting to the convolutional neural network (CNN) to deduce feature representations to distinctively detect seizures from the videos. The experimental results from different epileptic seizure types show a supreme performance of up to 78.33% AUC value, which demonstrates the promising prospect to utilize deep learning as a tool for curing epilepsy.

Fang et al., 2015 explored the anatomical connectivity differences underlying functional variance. Based on the constructed anatomical networks, multivariate pattern analysis is applied to extract the anatomical connectivity differences between the left and right TLE patients. Cantor-Rivera et al., 2015 derived an accuracy rate of more than 82% by using clinical parameters and extracting features of MRI and DTI images to identify TLE. Though a great TLE diagnosis rate of more than 80% has been made in most studies, a significant amount of misdiagnosis remains (around 10–20%). Many normal people are often mistakenly identified with a correct diagnoses on TLE disease. Moreover, these samples are in a small range and it is unclear whether they can be directly applied to other hospitals or not. To solve it, current state-of-the-art methods consider utilizing the toolbox of machine learning (deep learning) to achieve a high classification of epilepsy patients and normal persons (Zafar et al., 2017; Ahmedt-Aristizabal et al., 2018; Guo et al., 2020), and analyzing the changes of functional connectivity between enhanced and weakened brain regions (Rajpoot et al., 2015). Most experimental data of these methods are collected from EEG, fMRI, EEG fMRI, etc. (Pedreira et al., 2014; Sarraf and Tofighi, 2016; Vergun et al., 2016). While the combinations with neural networks further reduces the possibility of misdiagnoses, the limitation remains unsolved: Though off-the-shelf approaches can identify epilepsy patients and normal subjects, they fail to tell the specific epilepsy subtypes.

In this paper, we investigate the classification of TLE subtypes by integrating the self-attention mechanism and multilayer perceptron based method on our collected MEG dataset, aiming to find out the functional connection and pathogenesis of the brain network related to the seizure of these two subtypes. This research is important to propose a more rapid, more accurate, and intelligent subtype recognition method. To that effect, our method, termed MSAM, builds a multi-head self-attention model to predict epileptic seizures, where the original MEG signal is fed as its input. The self-attention mechanism analyzes the influence of the position of the sampled signal, so as to set different weights for the classification algorithm. The pre-seizure and interictal periods are separated, and then the multilayer perceptron model is used to extract the information of frequency and time domain to realize the feature extraction and classification. We propose to construct a multi-head self-attention model and apply it to the temporal lobe epilepsy subtype recognition algorithm.

To summarize, our main contributions are:

• to investigate the characteristics of TLE subtypes;
• to propose an end-to-end multi-head self-attention model, called MSAM, that predicts TLE subtypes;
• to evaluate the proposed model on a real-world dataset with classification task, demonstrating that the MSAM is superior to all comparative methods.

## Materials and Methods

**FIGURE 1:**
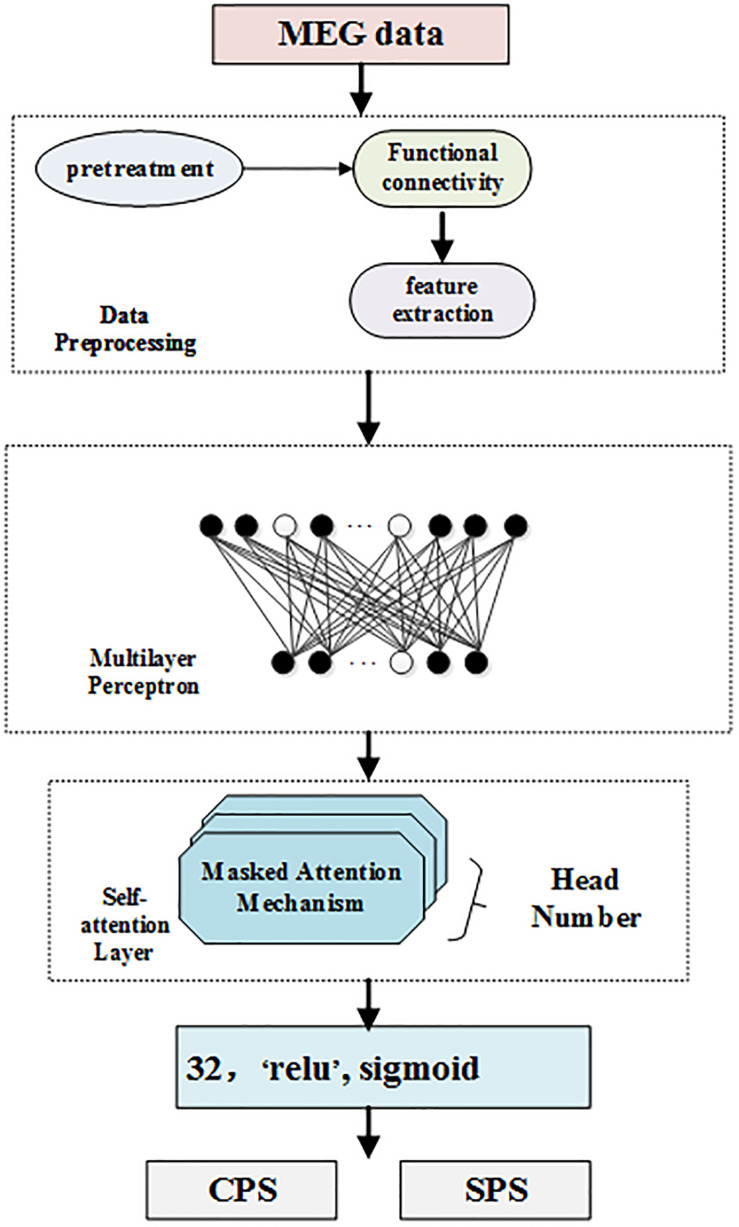
Multi-head self-attention neural networks.

In this section, we introduce our multi-head self-attention model to classify subtypes of epilepsy since the classification of epilepsy is more important to epilepsy physicians than the position of epilepsy. On one hand, the same detected discharge location may cause different symptoms for different patients. On the other hand, even though the clinical symptoms are the same, the positioning results may be completely different. Thus, the clinical symptoms, locations, and subtypes of epilepsy patients are very complicated. To solve this, in this paper, we propose to make full use of the self-attention mechanism to distinguish different symptoms in the same location. Meanwhile, we further adopt multilayer perceptron to solve the obstacle that the same clinical symptom possesses varying positions. Figure 1 displays the framework of our self-attention mechanism based deep learning network for epilepsy recognition.

We consider a set of *N* training dataset D = {(*t*_*i*_:*l*_*i*_), *i* = 1,…,*N*} where *t*_*i*_ is the *i − th* sample and *l*_*i*_ is its corresponding label. In our settings, *l*_*i*_ = 1 if the *i − th* sample is detected, and 0 if otherwise. Our network takes the *i − th* sample as its input and forward it to predict the label *l*_*i*_ As can be seen from Figure 1, our framework consists of four components: data preprocessing level, which processes MEG data; feature extraction, multilayer perceptron layer, which is a feed forward neural network; multi-head self-attention layer, which analyzes the weights of locations; and the last layer, which classifies and detects TLE subtypes.

### Multilayer Perceptron Layer

Multilayer perceptron (MLP) is a feed forward neural network model. MLP contains one dropout layer and four dense layers. The MLP module is shown in Figure 1. Each layer of the network is composed of multiple nodes. Except for the nodes in the output layer, each node is connected with all nodes in the next layer.

Dropout technology ensures that in the every iteration of the process for neural network training, dropout technology will randomly stop a certain number of neurons in the hidden layer, and use the mask process to set the output of these neurons in the hidden layer to 0, while the connection weights of the non-working neurons will not be updated in this iteration process. When the trained model is used in the test set, all nodes need to be used, and the neurons in the stopped-working state will return to work. Dropout technology effectively enhances the generalization ability of the deep neural network model and plays an important role in preventing over fitting of the deep learning model.

In Figure 1, the white nodes represent the neurons that will stop working according to a certain probability. After the dropout layer, there are four fully connected layers. The number of neurons in each hidden layer is 1024, 512, 128, and 32, respectively, corresponding to the activation functions of ELU, tanh, tanh, and relu.

### Self-Attention Layer

Self-attention is an attention mechanism relating different positions of a single input sequence to compute a representation of the same sequence. In order to obtain these representations, every input is multiplied with a set of weights for Keys (denoted as K), a set of weights for queries (denoted as Q), and a set of weights for values (denoted as V). Then, the self-attention learns a function mapping query Q to a series of key-value pairs (K, V), as follows:

(1) $$A\,t\,t\,e\,n\,t\,i\,o\,n\,V=Q\,K^{T}\,V$$

Attention essentially assigns a weight coefficient to each element in the sequence, which can also be understood as soft addressing. If each element is stored, the attention can calculate the similarity between Q and K. The similarity calculated by Q and K reflects the importance of the extracted *V* value, that is, the weight, and then the weighted summation obtains the attention value. The special point of the Self Attention mechanism in the K, Q, V model is that *Q* = K = V:

(2) $$\mathrm{Attention}\,\left(Q,K,V\right)=\text{softmax}\,\left(\frac{{\text{QK}}^{\text{T}}}{\sqrt{{\text{d}}_{\text{K}}}}\right)\,V.$$

The multi-head attention mechanism obtains *h* (i.e., one per head) different representations of (*Q, K, V*), computes self-attention for each representation, and concatenates the results. This can be expressed in the same notation as Eq. (4):

(3) $${\text{head}}_{i}=\text{Attention}\,\left(Q\,W_{i}^{Q},K\,W_{i}^{k},V\,W_{i}^{v}\right)$$

(4) $$\text{MultiHead}\left(Q,K,V\right)=\text{Contact}\left(head_{1}\ldots .,head_{h}\right)W^{0}$$

where the projections are parameter matrices $W_{i}^{Q}\,\varepsilon \,{\mathbb{R}}^{d\times d_{k}}$, $W_{i}^{k}\,\varepsilon \,{\mathbb{R}}^{d\times d_{k}}$, $W_{i}^{v}\,\varepsilon \,{\mathbb{R}}^{d\times d_{v}}$, and *W*^o^ε*ℝ*^*hd*_*v*_×*d*^, *d*_*k*_ = *d*_*v*_ = *d*/*h*.

### Classification and Detection Layer

To correctly predict if a sample is detected, we further deploy a softmax layer on top of the neural network. The basic process of the softmax layer is to map the output representation of the encoding layer into a probability interval (0, 1). In this paper, we regard the detection as a binary classification problem. Then, we forward input samples to the encoding network, outputs of which are further mapped into the probability interval (0, 1) by the softmax layer as below:

(5) $$l_{i}=\text{P}\,\left(t_{i}|S_{i}\right)=\frac{1}{1+e^{-\left(W_{c}\,u+b_{c}\right)}}\,\varepsilon \,\left(0,1\right)$$

W_*c*_ is the weight matrix and b_*c*_ is the bias term. Finally, we use cross-entropy between the ground truth visit *y*_*i*_ and the predicted visit *Y*_*i*_ to calculate the loss for each patient from all the timestamps as below:

(6) $$\text{L}\left(\theta \right)=-\frac{1}{\text{N}}\sum_{i=1}^{\text{N}}\left(y_{i}^{\text{T}}\log\left({\overset{⌢}{y}}_{t}\right)+{\left(1-y_{t}\right)}^{\text{T}}\log\left(1-{\overset{⌢}{y}}_{t}\right)\right)$$

### MEG Data

We collected our MEG data by recording 32 epilepsy patients from Brain Hospital Affiliated to Nanjing Medical University, China. To ensure the balance of data distribution, half of them were males and the other half were females. The age range of these patients varies from 20 to 32. The Institutional Review Board was approved and written consent was obtained from all subjects.

In more detail, the sampling frequency of our MEG data is 1200 Hz, and the signals have been filtered by the band-pass filter (0.03∼300 Hz), which is then digitized at 1000 Hz. We collected at least 20 groups of data for each subject and every group was observed for 2 min. That is to say, the total duration of the MEG raw data on each subject was no less than 40 min. The distance of head movement before and after MEG data collection was also measured, then those with a distance greater than 5 mm were discarded and re-measured to ensure the quality of collected data. In the process of data collection, both audio and video systems were used to monitor the subjects constantly. Moreover, the subjects were requested to be in a supine position with their eyes closed and to keep relaxed, such that the resting-state data could be observed.

### Evaluation Index

To evaluate the performance index, we first built the confusion matrix, upon which we further calculated the number of true-positive samples (TP), true-negative samples (TN), false-positive samples (FP), and false-negative (FN) samples.

To deal with the task of recognizing TLE subtypes, including CPS and SPS, four evaluation metrics are considered: precision (denoted as P), accuracy (denoted as ACC), recall (denoted as R), and the F1 score. In more detail, precision can be defined as:

(7) $$p=\frac{T\,P}{T\,P+F\,P}$$

Accuracy is expressed as the ratio of the number of correctly classified samples and the total number of samples on the test data set:

(8) $$\mathrm{ACC}=\frac{T\,P+T\,N}{T\,P+T\,N+F\,P+F\,N}$$

Recall rate can be formulated as:

(9) $$R=\frac{T\,P}{T\,P+F\,N}$$

F1 value is the harmonic mean of precision rate and recall rate, which can be rewritten as:

(10) $$\frac{2}{F\,1}=\frac{1}{P}+\frac{1}{R}\,\mathrm{F1}=\frac{2\,T\,P}{2\,T\,P+F\,P+F\,N}$$

As can be seen, a higher accuracy metric will lead to a better F1 score. Generally, the accuracy indicates the correct number of positive predictions. Recall represents the number of prediction-correct positive cases, which is directly related to the true-positive (TP) samples and false-negative samples (FN).

### Compared Methods

To show the effectiveness of our proposed MSAM, we compare our method with other models including Convolutional Neural Network (CNN), Support Vector Machine (SVM), and Random Forest (RF). CNN is a conventional deep learning model used to classify TLE subtypes, and SVM and RF are traditional machine learning algorithms. More details about these models are provided below:

Convolutional Neural Network (CNN): CNN is a kind of feed forward neural network with a deep structure using convolution computation. It is one of the most representative algorithms in deep learning.

Support Vector Machine (SVM): SVM is a kind of generalized linear classifier that classifies data using the supervised labels. Its decision boundary is the maximum margin hyperplane.

Random Forest (RF): RF is a classifier which contains multiple decision trees, and the output category is determined by the category output of individual trees.

## Results

### Performance Comparison

For fair comparison, all methods, including the proposed MSAM and the compared CNN, SVM, and RF, are trained with 300 epochs with a batch size of 32 and the results are calculated using cross-validation across the entire dataset. The head number in the self-attention of our method is set to 4.

We report the experimental results in Table 1. As can be seen from the table, the performance of our proposed MSAM takes a lead position in comparison with others. Specifically, it increases the performance of CNN by 0.4% on accuracy, 1.1% on recall, 0.7% on precision, and 1.1% on F1-score. Also, it outperforms SVM by 28.4, 75.5, 35.8, and 15.2% on accuracy, recall, precision, and F1-score, respectively. Besides, 1.0, 21.9, 13.0, and −1.8% gains are, respectively, obtained w.r.t accuracy, recall, precision, and F1-score on the basis of RF. The above experiments well demonstrate the capacity of our method in dealing with the MEG data classification.

**TABLE 1:** Comparison of CNN, SVM, RF, and the proposed MSAM.

**Method**	**Accuracy**	**Recall**	**Precision**	**F1-score**
CNN	0.832	0.898	0.9	0.823
SVM	0.552	0.154	0.549	0.682
RF	0.826	0.69	0.777	0.852
MSAM	0.836	0.909	0.907	0.834

### Effect of Varying Head Number in Self-Attention

**FIGURE 2:**
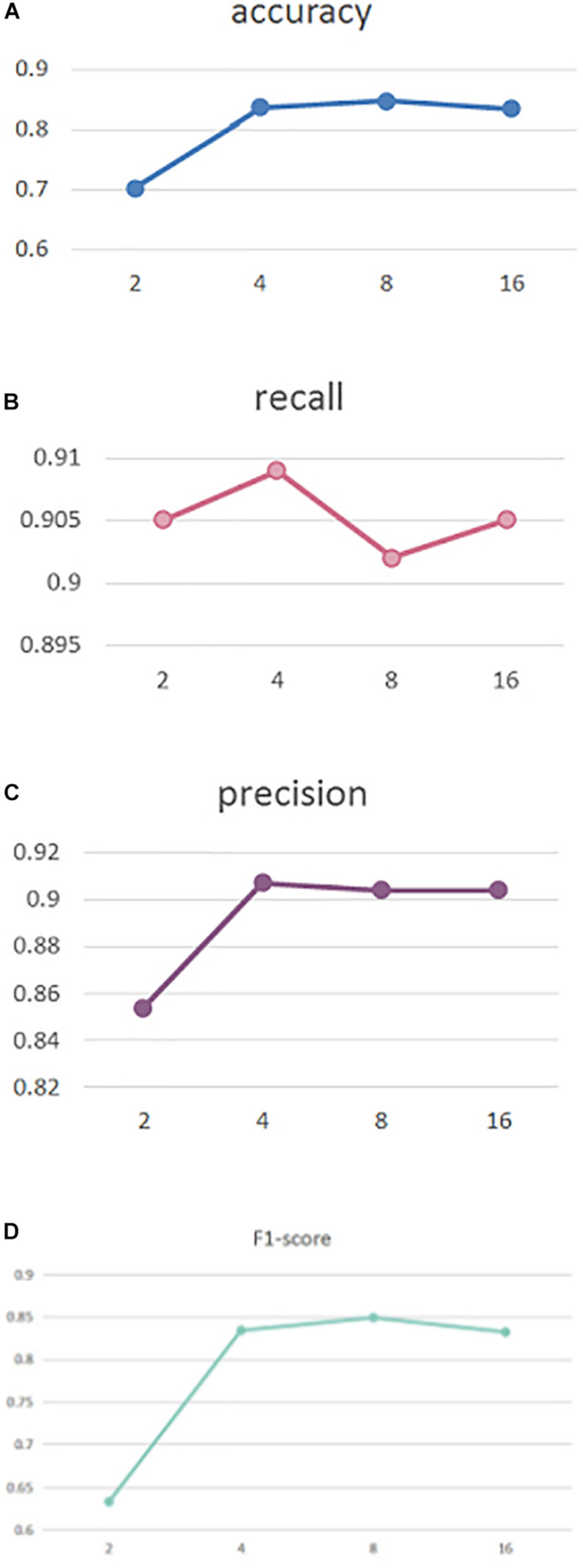
Performance comparison **(A)** Accuracy, **(B)** Recall, **(C)** Precision, and **(D)** F1-score.

To explore the effect of head number in our self-attention, in Figure 2 we perform experiments with different head numbers of 2, 4, 8, and 16. Similarly, the results are calculated using cross-validation across the entire dataset Figures 2A–D, respectively, display our performance of accuracy, recall, precision, and F1-score with different head numbers.

As can be seen, our MSAM obtains its best accuracy and F1-score performance when the head number is 8, while best recall and precision was obtained with a head number of 4. To reduce the model complexity, in our implementations we set the head number to 4.

### Ablation Study

We further conducted experiments to analyze the contributions of different components in our proposed method, including the multi-head self-attention layer and the self-attention layer. By, respectively, removing these two components, we have the following testing scenarios:

(1) Atten_1: The self-attention layer is removed;
(2) Atten_2: The multi-head self-attention layer is removed;
(3) MSAM: Both components are preserved, which makes up our method.

Following the above experimental settings, we train all models with 300 epochs and a batch size of 32. The head number is set to 4.

Table 2 shows the testing results. We can observe that our MSAM obtains the best performance, which demonstrates that both components of multi-head self-attention layer and the self-attention layer are crucial in boosting our classification performance.

**TABLE 2:** Comparison of ablated models.

**Method**	**Accuracy**	**Recall**	**Precision**	**F1-score**
attn_1	0.827	0.854	0.864	0.832
attn_2	0.825	0.845	0.859	0.831
MSAM	0.836	0.909	0.907	0.834

## Discussion

To correctly classify the subtype of TLE, including CPS and SPS, is very important to the treatment of patients. Most existing studies focus on distinguishing if one person suffers from epilepsy while ignoring the importance of which type of epilepsy the patient is suffering from. To solve this, in this paper, we developed a deep learning-based classification model by integrating self-attention mechanism to enhance the classification of TLE subtypes.

To this end, our proposed MSAM model is performed on our collected MEG data from 32 patients, made up of 16 males and 16 females aged from 20 to 32. As shown in Table 1, the proposed MSAM significantly outperforms its counterparts, including CNN, SVM, and RF, with a supreme performance of 83.6% in accuracy, 90.9% on recall, 90.7% on precision, and 83.4% on F1-score. Thus, our method can be well applied to the problem of classifying the subtypes of TLE.

By setting head numbers to 2, 4, 8, and 16, we analyze the effect of different head numbers in Figure 2, which shows that head number of 4 and 8 rewards the best performance. In the experiments, to reduce the model complexity, we set it to 4.

We also conducted an ablation study to explore the efficacy of different components in our method. The experiments in Table 2 show that the self-attention layer brings gains of 0.9, 5.5, 4.3, and 0.2% on accuracy, recall, precision, and F1-score, respectively. Besides, the multi-head self-attention layer also increased the performance by 1.1% on accuracy, 6.4% on recall, 4.8% on precision, and 0.3% on F1-score. Thus, both components of our MSAM play an important role in the classification of TLE subtypes.

Though significant contributions were made, limitations remain in this paper. First, the experiments were conducted on our collected single data source. More experiments on other datasets might be necessary to test the ability of the classification model. Second, epilepsy is a dynamically changed process. However, our classification focuses on patients from the same period, which may impede its practical applications. A long-term tracking experiment is needed. Third, this paper lacks studies on the prevention and early treatment of temporal lobe epilepsy. Due to the limited resources, we could not solve the limitations completely, which would be our focus in future work.

To summarize, this paper discusses and analyzes the classification of TLE subtypes. By integrating a self-attention mechanism, our MSAM is proposed to offer an effective classification model. The experimental results well demonstrate the effectiveness of our MSAM in classifying the TLE subtypes of CPS and SPS. Further works will be done to implement the limitations of this work as discussed above.

## Data Availability Statement

The raw data supporting the conclusions of this article will be made available by the authors, without undue reservation. Requests to access these datasets should be directed to TW, lvwu123@163.com.

## Ethics Statement

The Institutional Review Board approved the study with human subjects and the written consents were obtained from all subjects.

## Author Contributions

PG, TW, LZ, YP, JG, and JX conceived and designed the experiments. LZ, YP, JM, XP, MZ, and JG performed the experiments. LZ, PG, HL, MZ, and JG wrote the manuscript. All authors contributed to the article and approved the submitted version.

## Conflict of Interest

The authors declare that the research was conducted in the absence of any commercial or financial relationships that could be construed as a potential conflict of interest.

## References

[B1] Achilles F. Tombari F. Belagiannis V. Loesch A. M. Noachtar S. Navab N. (2018). Convolutional neural networks for real-time epileptic seizure detection. *Comput. Methods Biomech. Biomed. Eng. Imaging Vis.* 6 264–269. 10.1080/21681163.2016.1141062

[B2] Ahmedt-Aristizabal D. Fookes C. Nguyen K. Sridharan S. (2018). “Deep classification of epileptic signals,” in *Proceeding of the 40th Annual International Conference of the IEEE Engineering in Medicine and Biology Society*, Honolulu, HI, 332–335. 10.1109/embc.2018.8512249 30440405

[B3] Anastasiadou M. N. Christodoulakis M. Papathanasiou E. S. Papacostas S. S. Hadjipapas A. Mitsis G. D. (2019). Graph theoretical characteristics of EEG-based functional brain networks in patients with epilepsy: the effect of reference choice and volume conduction. *Front. Neurosci.* 13:221. 10.3389/fnins.2019.00221 30949021 PMC6436604

[B4] Beghi E. Giussani G. (2018). Aging and the epidemiology of epilepsy. *Neuroepidemiology* 51 216–223. 10.1159/000493484 30253417

[B5] Burns S. P. Santaniello S. Yaffe R. B. Jouny C. C. Crone N. E. Bergey G. K. (2014). Network dynamics of the brain and influence of the epileptic seizure onset zone. *Proc. Natl Acad. Sci. U.S.A.* 111 E5321–E5330. 10.1073/pnas.1401752111 25404339 PMC4267355

[B6] Cantor-Rivera D. Khan A. R. Goubran M. Mirsattari S. M. Peters T. M. (2015). Detection of temporal lobe epilepsy using support vector machines in multi-parametric quantitative MR imaging. *Comput. Med. Imaging Graph.* 41 14–28. 10.1016/j.compmedimag.2014.07.002 25103878

[B7] Englot D. J. Nagarajan S. S. Wang D. D. Rolston J. D. Mizuiri D. Honma S. M. (2016). The sensitivity and significance of lateralized interictal slow activity on magnetoencephalography in focal epilepsy. *Epilepsy Res.* 121 21–28. 10.1016/j.eplepsyres.2016.01.009 26871959 PMC4769925

[B8] Fang P. An J. Zeng L.-L. Shen H. Chen F. Wang W. (2015). Multivariate pattern analysis reveals anatomical connectivity differences between the left and right mesial temporal lobe epilepsy. *Neuroimage Clin.* 7 555–561. 10.1016/j.nicl.2014.12.018 25844312 PMC4375640

[B9] Guo J. Li H. Pan Y. Gao Y. Sun J. Wu T. (2020). Automatic and accurate epilepsy ripple and fast ripple detection via virtual sample generation and attention neural networks. *IEEE Trans. Neural Syst. Rehabil. Eng.* 28 1710–1719. 10.1109/tnsre.2020.3004368 32746301

[B10] Jette N. Fiest K. M. Sauro K. M. Wiebe S. Patten S. B. (2017). Author response: prevalence and incidence of epilepsy: a systematic review and meta-analysis of international studies. *Neurology* 89 641–642. 10.1212/wnl.0000000000004206 28784639

[B11] Liu J. Sun S. Liu Y. Guo J. Li H. Gao Y. (2020). A novel megnet for classification of high-frequency oscillations in magnetoencephalography of epileptic patients. *Complexity* 2020:9237808 10.1155/2020/9237808

[B12] Mariani V. Revay M. D’Orio P. Rizzi M. Pelliccia V. Nichelatti M. (2019). Prognostic factors of postoperative seizure outcome in patients with temporal lobe epilepsy and normal magnetic resonance imaging. *J. Neurol.* 266 2144–2156. 10.1007/s00415-019-09394-x 31127383

[B13] Niu K. Guo J. Pan Y. Gao X. Peng X. Li N. (2020). Multichannel deep attention neural networks for the classification of autism spectrum disorder using neuroimaging and personal characteristic data. *Complexity* 2020:1357853 10.1155/2020/1357853

[B14] Pedreira C. Vaudano A. E. Thornton R. C. Chaudhary U. J. Vulliemoz S. Laufs H. (2014). Classification of EEG abnormalities in partial epilepsy with simultaneous EEG-fMRI recordings. *Neuroimage* 99 461–476. 10.1016/j.neuroimage.2014.05.009 24830841

[B15] Peng X. Long G. Shen T. Wang S. Jiang J. (2020a). Self-attention enhanced patient journey understanding in healthcare system. *arXiv* [Preprint].

[B16] Peng X. Long G. Shen T. Wang S. Jiang J. Blumenstein M. (2019). “Temporal self-attention network for medical concept embedding,” in *Proceedings of the 19th IEEE International Conference on Data Mining, Beijing*, eds Wang J. Shim K. Wu X. (Piscataway, NJ: IEEE), 498–507.

[B17] Peng X. Long G. Shen T. Wang S. Jiang J. Zhang C. (2020b). BiteNet: bidirectional temporal encoder network to predict medical outcomes. *arXiv* [preprint].

[B18] Rajpoot K. Riaz A. Majeed W. Rajpoot N. (2015). Functional connectivity alterations in epilepsy from resting-state functional MRI. *PLoS One* 10:e0134944. 10.1371/journal.pone.0134944 26252668 PMC4529140

[B19] Sarraf S. Tofighi G. (2016). “Deep learning-based pipeline to recognize Alzheimer’s disease using fMRI data,” in *Proceedings of the 2016 Future Technologies Conference (FTC)*, San Francisco, CA.

[B20] Serna J.A.d.l.O Arrieta Paternina M. R. Zamora-Mendez A. Tripathy R. K. Pachori R. B. (2020). EEG-rhythm specific taylor-fourier filter bank implemented with o-splines for the detection of epilepsy using EEG signals. *IEEE Sens. J.* 20 6542–6551. 10.1109/jsen.2020.2976519

[B21] Shi Q. Zhang T. Miao A. Sun J. Sun Y. Chen Q. (2020). Differences between interictal and ictal generalized spike-wave discharges in childhood absence epilepsy: a MEG study. *Front. Neurol.* 10:1359. 10.3389/fneur.2019.01359 32038453 PMC6992575

[B22] Siuly S. Li Y. (2015). Designing a robust feature extraction method based on optimum allocation and principal component analysis for epileptic EEG signal classification. *Comput. Methods Programs Biomed.* 119 29–42. 10.1016/j.cmpb.2015.01.002 25704869

[B23] van Klink N. Zijlmans M. (2019). High frequency oscillations in MEG: next steps in source imaging for focal epilepsy. *Brain* 142 3318–3320. 10.1093/brain/awz321 31665753

[B24] Vergun S. Gaggle W. Nair V. A. Suhonen J. I. Birn R. M. Ahmed A. S. (2016). Classification and extraction of resting state networks using healthy and epilepsy fMRI data. *Front. Neurosci.* 10:440. 10.3389/fnins.2016.00440 27729846 PMC5037187

[B25] Wiebe S. (2013). Definition of drug-resistant epilepsy: is it evidence based? *Epilepsia* 54 9–12. 10.1111/epi.12176 23646963

[B26] Wu T. Chen D. Chen Q. Zhang R. Zhang W. Li Y. (2018). Automatic lateralization of temporal lobe epilepsy based on MEG network features using support vector machines. *Complexity* 2018, 1–10 10.1155/2018/4325096

[B27] Yao J. Wang H. Xiao Z. (2019). Correlation between EEG during AED withdrawal and epilepsy recurrence: a meta-analysis. *Neurol. Sci.* 40 1637–1644. 10.1007/s10072-019-03855-x 31011931

[B28] Zafar R. Malik A. S. Shuaibu A. N. Rehman M.J.u Dass S. C. (2017). “Classification of fMRI data using support vector machine and convolutional neural network,” in *Proceedings of the 2017 IEEE International Conference on Signal and Image Processing Applications, Kuching*, (Piscataway, NJ: IEEE), 324–329.

